# A Systematic Review and Meta-Analysis of the Effects of Various Sources and Amounts of Copper on Nursery Piglets

**DOI:** 10.3390/vetsci11020068

**Published:** 2024-02-02

**Authors:** Pedro Augusto Galiotto Miranda, Aline Remus, Danyel Bueno Dalto, Rafaela Hilgemberg, Guilherme Beber Jasluk, Brena Cristine Rosário Silva, Cheila Roberta Lehnen

**Affiliations:** 1 Department of Animal Science, Universidade Estadual de Ponta Grossa, Ponta Grossa 84030900, PR, Brazil; pedroagmiranda@gmail.com (P.A.G.M.); hilgembergrafaela@gmail.com (R.H.); guilherme.jasluk@gmail.com (G.B.J.); 2 Sherbrooke Research and Development Centre, Agriculture and Agri-Food Canada, Sherbrooke, QC J1M 0C8, Canada; aline.remus@agr.gc.ca (A.R.); danyel.buenodalto@agr.gc.ca (D.B.D.); 3 Department of Animal Science, Universidade Estadual de Maringa, Maringa 87020900, PR, Brazil; brena.silva@gmail.com

**Keywords:** copper level, copper source, meta-analysis, performance, pig

## Abstract

**Simple Summary:**

Supranutritional levels of copper with both inorganic and organic sources are commonly used as growth promoters for nursery piglets, but different responses are observed between studies. Therefore, this study uses a combination of systematic review and meta-analysis to evaluate the effects of supranutritional levels and sources of copper on the performance of nursery piglets. Our results indicate that, regardless of the sources used in the diet, the best growth performance results for nursery piglets were detected with dietary levels between 80 and 200 mg Cu/kg.

**Abstract:**

This study evaluated the impact of different dietary levels and sources of copper on the growth performance of nursery piglets through a combination of systematic review and meta-analysis. The database for this study was created using articles selected from major electronic databases. Data analysis involved forest plots and analysis of variance using mixed-effects models. The database included 63 articles published between 1990 and 2021, comprising 21,113 piglets in 946 treatments. Positive effects of supranutritional levels of copper from both inorganic and organic sources on the growth performance of nursery piglets were detected using Forest plots and analysis of variance (*p* < 0.001). Using mixed models, it was observed that piglet performance is influenced by body weight (*p* < 0.001), age (*p* < 0.001), and copper intake (*p* < 0.001). Both organic and inorganic sources of copper at supranutritional levels (>81 mg Cu/kg of diet) improved the performance of nursery piglets, but levels higher than 201 mg Cu/kg of diet did not further improve growth performance compared to 80–200 mg Cu/kg of diet. The feed conversion was worse in piglets fed with inorganic Cu sources (*p* < 0.001). In conclusion, dietary Cu supplementation influenced the weight gain and feed conversion rate in weaned piglets, particularly during the first few weeks post-weaning. Levels of 81 and 200 mg Cu/kg improved growth performance, but no further benefits were obtained for higher levels.

## 1. Introduction

High levels of dietary copper (Cu) are currently used as a growth promoter for nursery piglets because of their effects on improving weight gain and feed efficiency and reducing the occurrence of diarrhea in the first few weeks post-weaning [1]. Supplemented levels are viable and may be as high as 250–500 mg Cu/kg of diet [2], which exceeds the nutritional requirements of 5 to 15 mg/kg needed by piglets by a substantial amount [3]. Although high levels of dietary Cu have potential bactericidal functions capable of controlling pathogens at the intestinal level [4,5], such high levels lead to accumulation of this trace mineral in pig slurry, creating potential environment concerns [6] and contributing to the development of antimicrobial resistance to bacteria in the pig gut [7]. In the European Union, supplementary Cu doses in pig diets are limited to 170 mg/kg, with recommendations for the inclusion of sources with higher bioavailability [8].

Inorganic sources such as Cu sulfate (CuSO4) are more commonly used by the swine industry [9], but organic sources such as Cu bound to peptides or polysaccharides show higher intestinal absorption rates and retention in tissues [10]. Many studies have been conducted to evaluate the effect of different Cu sources on the performance of nursery piglets [6,10,11] and, although both sources appear to positively impact performance when supplemented at supranutritional levels, it is still not clear which is the most appropriate dose or source of copper for post-weaning piglets.

Meta-analysis allows us to group and compare different studies and estimate pooled means, enabling the generation of new hypotheses, which could be limiting in individual studies [12,13,14]. By using this approach, other studies evaluated supranutritional levels of Zn [15] and plasmatic Zn and Cu relations [16] for nursery piglets. Additionally, the effect of Zn inorganic sources for growing–finishing pigs in terms of growth performance and carcass quality [17] were evaluated. Moreover, the digestibility of Zn and Cu in pigs [18] and the impact of dietary Cu on broilers’ growth performance [19] were recently estimated. However, some limitations, such as the limited number of studies and the temporal space of publications in the databases, call for a new meta-analysis to evaluate different sources of Cu on the growth performance nursery piglets.

Therefore, the aim of the present study is to use a meta-analytical approach to integrate the available literature on dietary supplementation with different Cu dietary levels and sources and quantify their effects on the growth performance of nursery piglets.

## 2. Materials and Methods

### 2.1. Systematic Review and Database Compilation

A thorough systematic analysis was conducted to select the studies included in the database. The studies were primarily chosen from the main electronic databases (Pub Med, Scholar Google, Science Direct, Web of Science, Periódicos Capes, Scielo, Highwire Press), using search terms in Portuguese, English, and Spanish. The search strategy followed the adapted PICO method, combining and varying terms to define the population (pigs, nursery piglets), intervention (chelated Cu, organic Cu, inorganic Cu, Cu sulfate), and outcome (performance, weight gain, and feed intake). No temporal limitations were imposed during the literature search.

Following the selection criteria, studies were meticulously evaluated for their quality and relevance to the proposed objectives before being included in the database. The assessed criteria were (a) studies involving nursery piglets; (b) supplementation of organic and/or inorganic sources of dietary Cu; (c) growth performance responses (average daily gain, average daily feed intake, and feed conversion ratio). Studies that did not meet the above-described criteria were excluded from the database. Studies that met the selection criteria but presented results only in the form of graphs or figures were excluded as well. The significance level (*p*-value) for the effect of Cu sources in the studies was not considered a criterion for inclusion or exclusion in the eligibility analysis.

Following the selection process, the studies were included in a Microsoft Excel electronic spreadsheet in which each column represented a variable and each row represented a treatment. The tabulated data included information related to bibliographic details (authors, year, journal, country, and institution of origin, etc.), experimental characteristics (genetic line, age, initial body weight, experiment duration, housing temperature, nutritional feed composition, presence or absence of health challenge, type of challenge, pathogens, limiting nutrients, etc.), treatments (sources and Cu dietary levels), and growth performance results. The use of feed additives such as enzymes, antibiotics, or organic acids as performance enhancers was excluded in the tabulation.

### 2.2. Database Coding and Structuring

The definition of dependent and independent variables and the data coding were performed to enable the analysis of inter- and intra-experimental effects [12,13,20]. Moderating codes were created to consider (a) study effect, in which each article received a sequential number (COD 01, 02, 03...), (b) inter-study coding (COD article 01 + treatment = 011), and (c) intra-study coding, to consider the effects of repeated measurements over time. To characterize treatments and standardize groups in data analysis, studies were coded based on Cu source as basal diet (BD), inorganic and organic (COD BD, INO, and ORG) and Cu levels. Thus, to consider appropriate copper levels for nursery piglets, we consider grouping based on the literature [3,8]. Copper levels, regardless of the source used, were classified as follows: 1 to 15 mg Cu/kg diet was required to meet the nutritional requirements of piglets [3]; 16 to 80 mg Cu/kg diet was considered intermediate; 81 to 200 mg Cu/kg diet was considered supranutritional; and supplementations exceeding 201 mg of Cu/kg in the diet were considered highly supranutritional [3,8]. Most studies presented treatments containing different dietary levels of an organic or inorganic Cu source. Around 40% of studies contained a treatment with up to 15 mg Cu/kg of diet, and thus were considered BDs, along with increasing supranutritional Cu dietary levels which were then considered pharmacological for nursery piglets. Other studies presented treatments comparing organic and inorganic sources at different Cu levels. Other codes included classification for sources according to the Association of American Feed Control Officials [21], the presence of health challenges, piglet weaning age, sex, and genetic lineage.

The dietary composition was determined based on the original inclusion levels of each ingredient used in the respective study. Subsequently, the nutritional composition was recalculated using Evapig^®^ (v. 1.4.0.1; INRA, Saint-Gilles, France). This method was employed to minimize nutrient variations in diets across experiments (Table 1). The Cu levels in the BD were used as a reference to estimate the variation in the nutritional composition of each study. After this standardization, Zn and Cu intake were calculated based on the respective micromineral levels in the diets multiplied by the average daily feed intake for each treatment (Ingested Zn or Cu, mg/d). The Zn-to-Cu ratio was calculated by dividing the Zn by Cu levels presented in the experimental diets.

### 2.3. Statistical Analysis

Three statistical approaches were applied to explore the impact of dietary Cu for nursery piglets. (1) A comprehensive analysis using a forest plot, enabling a global view of the effects of Cu sources and levels; (2) analysis of variance using a mixed model to obtain adjusted means for growth performance; and (3) multiple regression mixed models which estimate and choose the best-fit models that explain the effects of Cu supplementation on piglet performance.

The comprehensive meta-analysis using forest plots displays the effects of copper supplementation in aggregated estimates for each study, the study’s contribution to the meta-analysis (weight), and the effect size of the sample (risk ratio) within the 95% confidence intervals [22]. In this analysis, the effects of organic and inorganic Cu were considered separately and the subgroups for levels 16–80 mg Cu/kg, 81–200 mg Cu/kg and >201 mg Cu/kg were analyzed. BD (1–15 mg Cu/kg) was considered as the control within each analysis. The heterogeneity, which estimates the diversity among studies using Higgins’ I2 method and Cochran’s Q, was considered low, moderate, and high with I2 values of 25%, 50%, and 75%, respectively [23]. The plots were created using the *metafor* package in R Studio 3.6.1. Regardless of intra-study variability (high heterogeneity), no study was excluded from forest plot analysis.

Analysis of variance and mixed-effects models were conducted to investigate other factors that potentially contribute to the heterogeneity among the studies. Prior to the analysis of variance, data were assessed for coherence and biological distribution, normality, and correlations. Correlation analysis between columns of the database was applied to identify the presence of collinearity between variables. In the statistical modeling, Cu sources and levels were treated as fixed effects, while the study was considered to have random effects. Covariates, including weaning age and initial body weight, were examined using Fischer’s test (*p* < 0.05) and incorporated into the statistical model. Least squares means of inter-experimental data for Cu sources and levels were calculated using analysis of variance, applying a generalized linear model with covariate adjustment and the study as random effect. Using the weaning age demonstrated a superior model fit and was chosen as covariate for the final model. The model initially included environmental temperature and dietary patterns as random effects; however, these variables, though important, were challenging to measure accurately and were consequently eliminated from the model. The effects of sex (male/female), genetic lines, and year of publication as fixed effects were found to be nonsignificant and were thus eliminated from the model. Moderating variables, such as the number of repetitions and the number of animals per experiment, were incorporated in the analysis of variance. Interactions between Cu source × levels were evaluated for all parameters. Unfortunately, due to limited data availability, interactions between weaning age and initial body weight were not measured. Comparisons between the data were made at the 5% significance level using Tukey’s test.

Multiple regression mixed models were developed to estimate and choose the best fit models for daily feed intake, average daily gain, and feed conversion of nursery piglets. Studies were treated as random effects and Cu sources and levels in the diet were treated as fixed effects. Data highly correlated with treatments were submitted, allowing the inclusion of various effects in the model, such as initial age, initial body weight, Zn and Cu intake, Zn/Cu ratio, and Cu sources. The data were analyzed using the lmer and summary functions in the *lmer4* package, generating relevant values for each model created, including its intercept. The performance package was utilized to evaluate the models and determine the best-fit model based on Akaike’s Information Criterion (AIC), Bayesian Information Criterion (BIC), Root Mean Square Error (RMSE), coefficient of determination (R2), and Intraclass Correlation Coefficient (ICC) for mixed-effects models [24]. Data analysis and visualization were performed using R Studio software (version 3.6.1; R Foundation for Statistical Computing, Vienna, Austria).

## 3. Results

### 3.1. Literature Search

**Figure 1:**
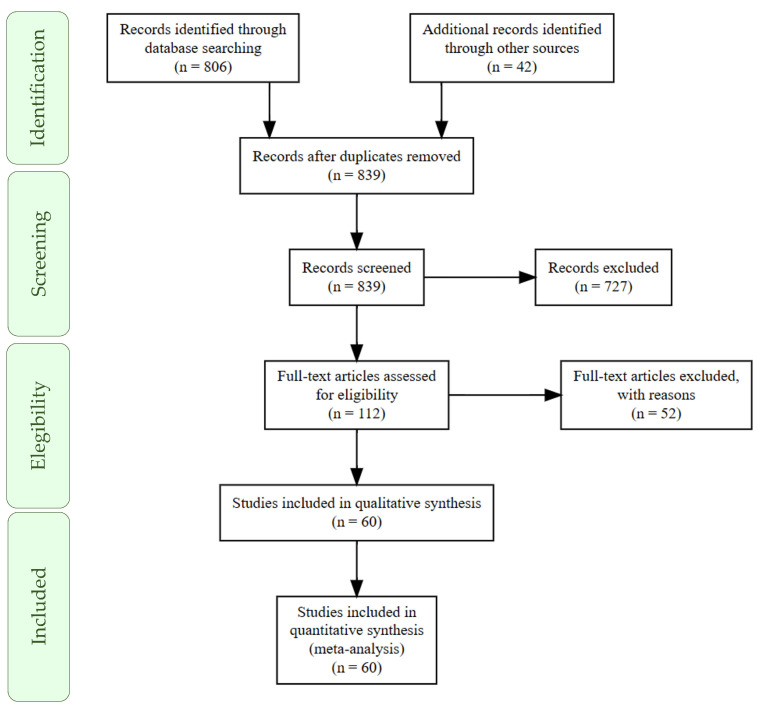
PRISMA flowchart describing the selection process of studies related to the effects of inorganic and organic copper sources in diets of weaned piglets.

The study’s temporal scope spanned 31 years (1990–2021). Considering the search strategy, approximately 112 articles published between 1990 and 2021 were initially selected for eligibility. After the analysis, 60 studies constituted the database, including 54 scientific articles and nine works from dissertations or theses, comprising 65 experiments. The PRISMA flowchart proposed by Moher et al. [25] illustrates the process of selecting studies for inclusion in the database (Figure 1).

### 3.2. Database Description

The database comprised 25,053 nursery piglets (on average, 272 animals per study), with body weights ranging from 7.0 kg (SD = 2.1) to 17.7 kg (SD = 7.3). The piglets were assessed at ages ranging from 28.1 days (SD = 9.0) to 49 days (SD = 13.6) and were distributed into 946 experimental groups. The experimental duration varied from a minimum of 10 days to a maximum of 60 days, with an average duration of 32.0 days. The temporal scope of the database spanned 31 years, with the oldest article published in 1990 and the most recent in 2021; the mode was 2011. The electronic Excel spreadsheet comprised 946 rows and 169 columns. Most studies were conducted in American institutions (44.44% of the articles), followed by Brazilian (20.63%), Chinese (17.5%), and Italian (3.05%) institutions. Among these treatments, 429 (45.35%) involved inorganic sources, 323 (34.14%) involved organic sources, and 194 treatments were part of the basal group (20.51%). Basal treatments had copper supplementation levels of up to 15 mg/kg of Cu in the diet. Nutritional levels (1 to 15 mg Cu/kg) were present in 194 treatments (20.51%), intermediate levels (16 to 80 mg Cu/kg) were observed in 167 treatments (17.65%), supranutritional levels (81 to 200 mg Cu/kg) were detected in 456 treatments (48.20%), and concentrations above 201 mg Cu/kg were present in 129 treatments (13.64%). A comprehensive description of the database employed in the meta-analysis of Cu sources and supplementation levels in nursery piglet diets is presented in Supplementary Table S1. The majority (78%) of the papers used hybrid piglets, 15% identified the piglets used as crossbreed and 63% identified the piglets used as being from specific genetic lines, whereas 21.6% did not report the piglets’ genetic lineage. Barrow/male and female piglets (mixed lots) accounted for 82% of the piglets, male piglets accounted for 15.8%, whereas 0.05% of the papers did not report any gender information.

### 3.3. Comphreensive Meta-Analysis

**Figure 2:**
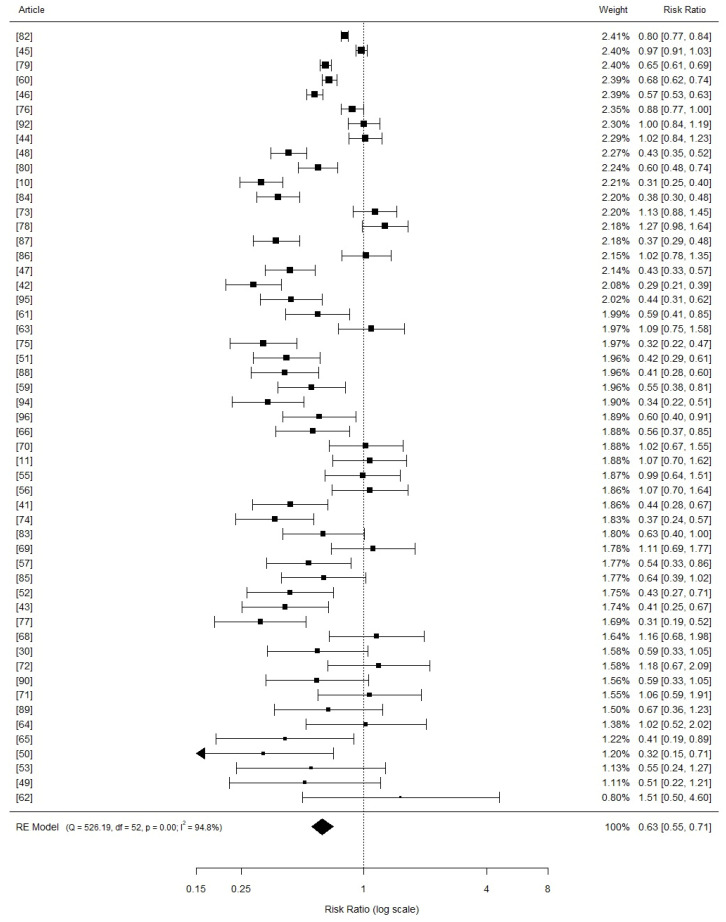
Forest plot (random effects model) depicting the results of a meta-analysis investigating the effects of inorganic copper on average daily gain in weaned piglets. Each square in the plot represents study-specific risk estimates, with the size of the square reflecting the specific statistical weight of the study. Horizontal lines indicate the 95% confidence intervals (CIs) for each study, and the diamond at the bottom of the plot signifies the summarized relative risk with its corresponding 95% CI.

Fifty-six studies containing inorganic copper sources were compared to the basal treatments in each study regarding the average daily gain outcome (Figure 2). The addition of inorganic copper had a positive effect on the weight gain of nursery piglets, as indicated by the pooled mean difference SMD (SMD = 0.63, 95% CI: 0.55 to 0.71, *p* = 0.000), regardless of the inclusion levels.

**Figure 3:**
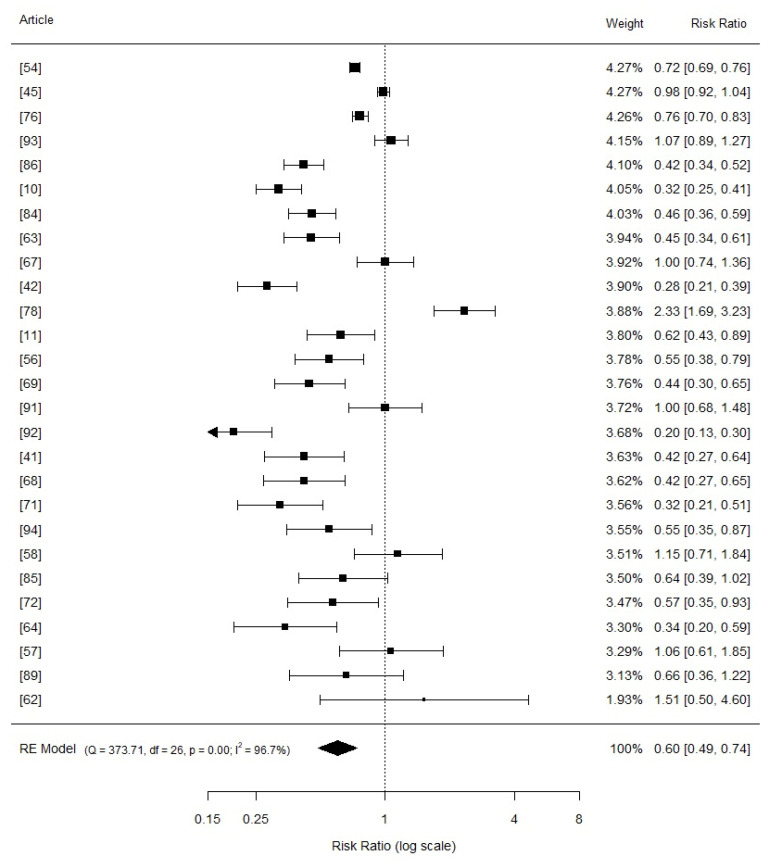
Forest plot (random effects) of the meta-analysis examining the effects of organic copper on average daily gain in weaned piglets. The squares in the plot represent study-specific estimates of the mean difference in average daily gain (the size of each square corresponds to the specific statistical weight of the study), while horizontal lines indicate the 95% confidence intervals (CIs). The diamond located at the bottom of the plot represents the summarized effect size (mean difference) with its associated 95% CI.

The addition of organic copper compared to the basal treatments involved 27 studies (Figure 3) and indicated a positive effect on the average daily gain of nursery piglets (SMD = 0.60, 95% CI: 0.49 to 0.74, *p* = 0.000), regardless of the inclusion levels. The high heterogeneity observed between the studies for both inorganic copper (I2 = 94.8%) and organic copper sources (I2 = 96.7%) was expected and can be explained by inherent intra-study factors in animal experimentation, such as animal and environmental characteristics, feeding, experimental design, and sample size.

**Figure 4:**
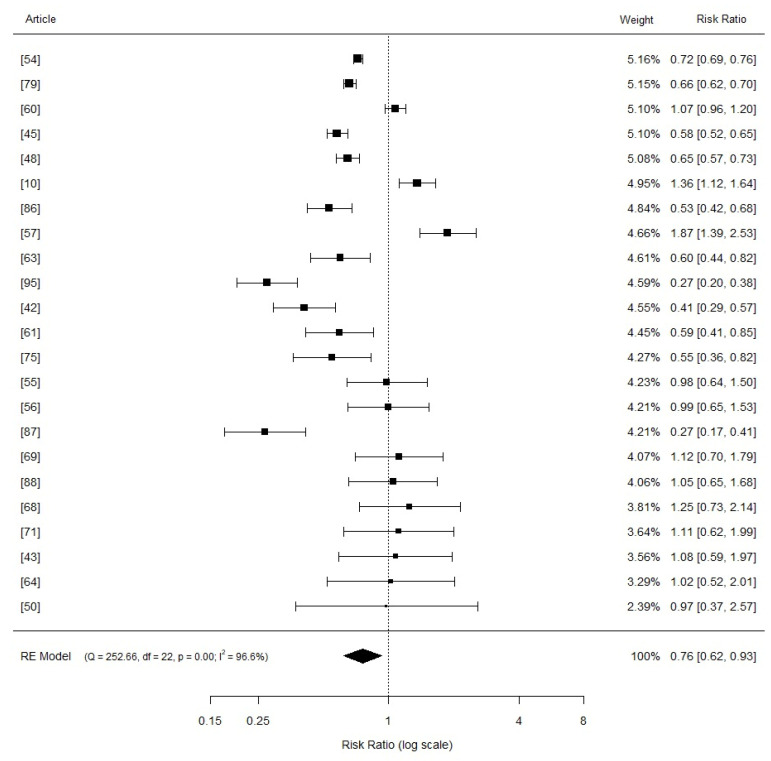
Forest plot (random effects model) of the meta-analysis regarding the effects of levels between 16 and 80 mg Cu/kg in diets compared to the basal diet (1–15 mg Cu/kg) on the average daily gain of weaned piglets. The squares represent study-specific estimates of the mean difference in average daily gain (the size of the square corresponds to the statistical weight of the study); horizontal lines depict 95% confidence intervals (CIs). The diamond at the bottom of the plot indicates the summarized effect size (mean difference) with its corresponding 95% CI.

**Figure 5:**
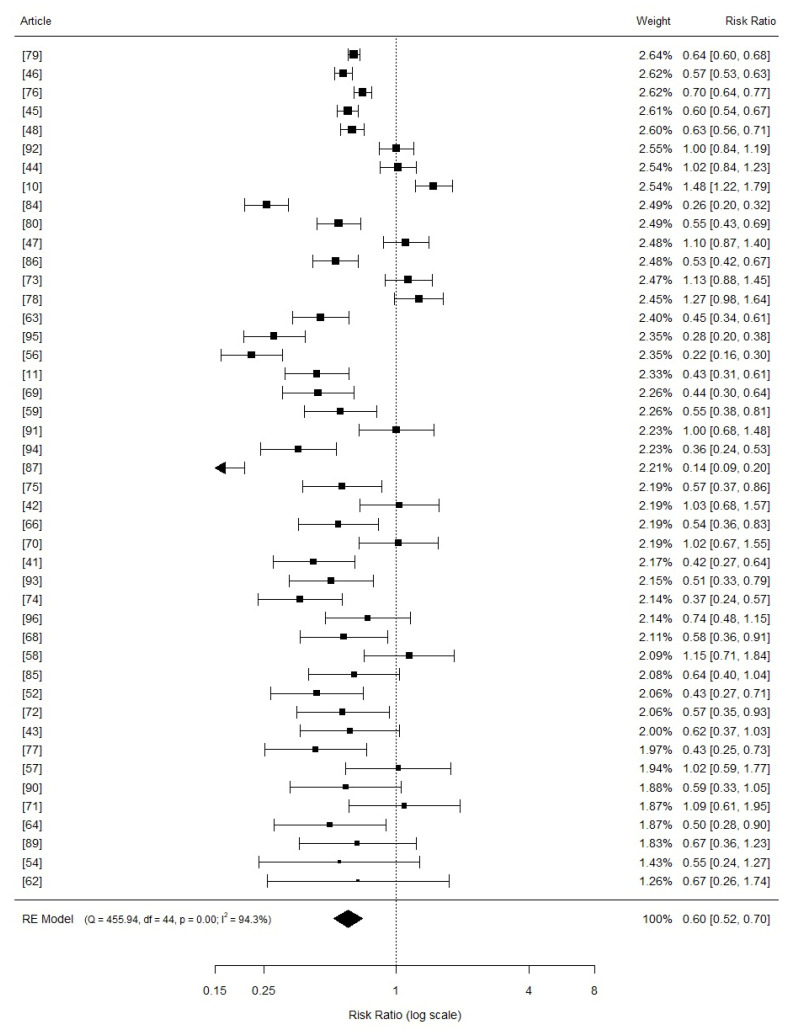
Forest plot (random model effects) illustrating the results of a meta-analysis examining the effects of copper levels between 81 and 200 mg/kg in diets compared to a basal diet with 1–15 mg/kg of copper on the average daily gain in weaned piglets. The squares represent study-specific estimates of the mean difference in average daily gain (the size of the square corresponds to the statistical weight of the study); horizontal lines depict 95% confidence intervals (CIs). The diamond at the bottom of the plot indicates the summarized effect size (mean difference) with its corresponding 95% CI.

**Figure 6:**
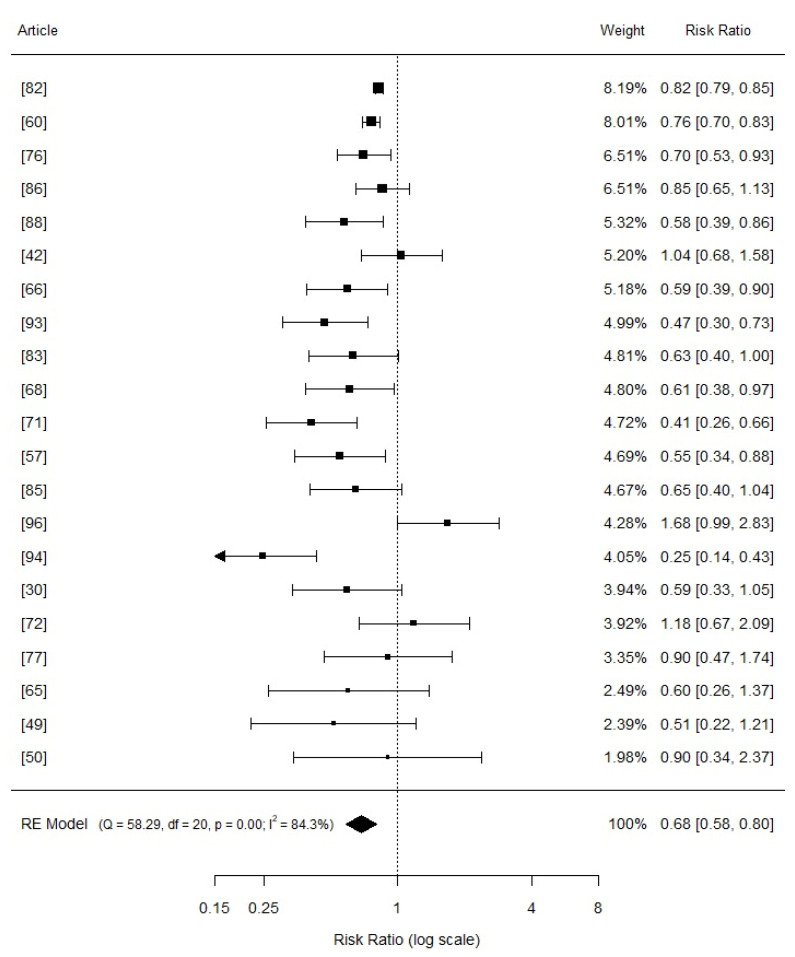
Forest plot (random effects) of the meta-analysis regarding the effects of levels between >201 mg Cu/kg in diets in relation to a basal diet (1–15 mg Cu/kg) on average daily gain in weaned piglets. The squares represent study-specific risk estimates (the size of the square reflects the specific statistical weight of the study); horizontal lines indicate 95% confidence intervals (CIs); the diamond indicates the summarized relative risk with its 95% CI.

When stratified by Cu levels, the pooled risk ratio for the average daily gain of nursery piglets within the 16–80 mg Cu/kg diet category, involving 23 studies, was 0.76 (95% CI: 0.62 to 0.93, *p* = 0.000; Figure 4). The risk ratio for the 81–200 mg Cu/kg diet category of nursery piglets was 0.60 (95% CI: 0.52 to 0.70, *p* = 0.000), comprising 45 studies (Figure 5). For diets with >201 mg Cu/kg in nursery piglets, involving 21 studies, the risk ratio was 0.68 (95% CI: 0.58 to 0.80, *p* = 0.000) (Figure 6). The Higgins index was 96.6%, 94.3%, and 84.3%, respectively.

### 3.4. Effects of Copper Supplementation on Piglets Growth Performance

In the variance analysis, we identified that weaning age directly influences piglet performance. This effect was included as a covariate, and the results were adjusted for the initial housing age. Both dietary Cu sources increased piglet average daily gain by 8% compared to the basal diet (*p* = 0.013, Table 2) and improved piglet feed conversion by 4% compared to piglets fed with the basal diet (*p* = 0.02). No difference was detected for any performance parameters for dietary copper levels between 81 and 200 mg/kg and >201 mg/kg (*p* < 0.05). Supranutritional levels >81 mg Cu/kg increased weight gain by 7% (*p* = 0.005) and decreased piglet feed conversion by 7% compared to levels <80 mg Cu/kg (*p* < 0.001). Copper sources and levels did not interfere with piglet feed intake (*p* > 0.05). There was no interaction between copper sources and levels for the analyzed variables.

Mixed-effects models allow the evaluation of factors that interfere with piglet performance and can explain the high heterogeneity in the forest plots meta-analysis (Table 3). Several mixed linear models were developed, but in this study, we present only the models that collectively showed the best AIC-, BIC-, R2-, RMSE-, and ICC-adjusted coefficients. Factors that did not indicate any statistical effects, such as sex, lineage, experimental design, sample size, among others, were excluded from the models.

In the models for feed intake, average daily gain, and feed conversion, the ingested copper, initial weight, and initial age were significant (*p* < 0.05). Ingested zinc and Zn/Cu ratio did not affect feed conversion (*p* > 0.05). The coefficient of determination (conditional R2) for the studies was high, with the model developed for feed intake explaining 73.7% of the variability in response data. In the models for weight gain and feed conversion, the coefficient of determination was 73.5% and 65.4%, respectively. The AIC for all three models developed was low, with the lowest value observed in Model 1 (−1600.6). The same trend was observed in the Bayesian Information Criterion (BIC), which describes the relationship between the dependent variable and the various explanatory variables among the models. Therefore, the lower the BIC, the better the model fit, similar to AIC. Model 1 shows the lowest results (−1553.3), although the other models also have similar BIC and AIC values. The residual variances of the models were small, as well as the Root Mean Square Error (RMSE), indicating that the models were accurate due to the low values found in the mentioned variables. We consider that the Intraclass Correlation Coefficient (ICC) in the models partially explains the relationships between the effects. In Model 2, nursery piglets exhibit a linear response for average daily gain (ADG), assuming that daily copper intake improves weight gain in the nursery by 0.0013 kg. In Model 3, we identified a slight worsening in feed conversion for piglets fed diets containing inorganic copper sources. The lack of significance of organic sources in the mixed models is probably associated with the smaller number of articles compared to inorganic sources.

## 4. Discussion

When comparing the meta-analysis approaches used, both the forest plot and the analysis of variance–covariance indicated similar trends. However, it is worth noting that the experimental design of each study should be carefully evaluated for use in a forest plot. The I^2^ values in both figures were above 50%, indicating high heterogeneity among the studies. In subgroup analysis, we identified that all Cu levels added to the diet, regardless of the source, positively influenced the average daily gain of nursery piglets. The heterogeneity tended to decrease, as the studies had similar methodological characteristics (experimental design, sources and doses, animal weight and genetics). Therefore, we chose to analyze the topic using more exploratory methods. Due to the experimental design, variability in piglet weight and age, and different Cu sources and levels in the diets, we considered it more appropriate, for the objective of this study, to analyze data using variance, covariance, and mixed models.

Cu inorganic sources, mainly Cu sulfate (CuSO4), are commonly supplemented in the different stages of pig production and have been used for several decades as growth promoters. Recently, due to increasing concerns about the environmental impact of livestock production, alternative sources with higher bioavailability, such as organic sources, have been sought. In our study, the main organic Cu sources, according to AFFCO [21] classification, were the chelates metal–amino acid complex, lysine, and methionine (30.27% of treatments). Other sources such as Cu proteinates (3.8%), organic metal–acid chelates (2.4%), and Cu complexed with polysaccharides (1.4%) were present in the database, but with a smaller number of treatments. Although it has been shown that Cu organic sources, especially chelates, may yield superior performance results compared to inorganic sources [10,26], in the present study, both sources enhanced piglets’ performance compared to the BD, but did not differ between them (Table 2). However, it is worth mentioning that at supranutritional doses (80–200 mg/kg), the growth performance results were more homogeneous across studies when using organic Cu, with fewer discrepancies compared to those observed when using inorganic forms. Although the explanation for these results may be outside the scope of this study, it can be argued that it is relevant due to the greater stability provided by the chelating agent [27]. Chelated minerals are more stable and less prone to reactions and interactions with other elements in the gastrointestinal tract [27].

In terms of the effects of copper levels, this meta-analysis indicates that high dietary Cu levels influence the performance responses of nursery piglets, particularly the average daily gain and feed conversion ratio. Nevertheless, regression models (linear, quadratic, or linear plateau models) were initially employed as the primary approach to estimate the optimal Cu level for piglet performance. Initial body weight, weaning age, and study effects were included in the models. However, the regression coefficients, characterized by low values, provided limited insight into the influence of Cu levels on average daily gain and feed conversion. Increasing levels of dietary Cu improved the daily weight gain and feed conversion ratio up to 200 mg Cu/kg, particularly in younger piglets (post-weaning), supporting the current Cu supplementation strategies using concentrations significantly higher than the NRC (2012) recommendations to enhance piglets’ performance during the nursery phase [28]. We identified that weaning age interferes with the response to Cu supplementation for nursery piglets. The effects of Cu supplementation appear to be more effective in younger piglets, independent of Cu sources or supranutritional levels. This result was verified by the variance and covariance analysis. However, no further benefits were detected with levels >200 mg/kg Cu or in older piglets (end of nursery). The mechanisms behind such effects are not yet fully understood. Some studies attributed these effects to local (intestine) antimicrobial functions of copper [1,29], whereas others [30,31,32] claim a systemic effect involving the interactions between copper and zinc. Although the present experimental approach does not allow any extrapolation of physiological and/or metabolic mechanisms, the mixed-models analysis indicate that Zn intake is indeed relevant to the effects of copper, irrespective of dietary Cu levels (see discussion below). Copper also has effects on energy metabolism, modulating both lipid absorption and lipogenesis [33], leading to an increase in fatty acid concentrations in the bloodstream [34] as well as ATP synthesis in the body [35]. These processes could potentially improve the animal’s energy efficiency, with implications for feed efficiency, as demonstrated by the present results. In terms of Cu’s effects on feed intake, it has been shown that Cu may modulate appetite, leading to increased feed intake [36].

A key factor interfering with Cu metabolism is Zn supplementation levels. The estimated average Zn supplementation in the present study was 665.4 mg/kg. The present mixed-models analysis (Table 3) indicated that the ingested Zn levels in piglet diets influence feed intake and average daily gain. Although restrictions on the use of high Zn levels in pigs’ diets have been imposed in many countries, pharmacological levels of dietary Zn are still used in some parts of the world. [2,37,38]. Although an accurate assessment of the inflexion point has yet to be performed, dietary levels between 1500 and 3000 mg/kg [39] can impair Cu metabolism due to the increased production and synthesis of metallothionein, a metalloprotein responsive to dietary Zn levels but with a greater affinity for copper. Under high dietary Zn supplementation, Cu is trapped by metallothionein within enterocytes impairing its release and further flow to the liver, eventually resulting in systemic copper deficiency [39]. According to Dalto et al. [40], a low dietary Zn/Cu ratio is desirable to avoid compromising piglets’ health with high systemic Zn concentrations and to avoid a potential Cu deficiency resulting from long-term high dietary Zn oxide during the post-weaning period.

Investigating the impact of Cu sources and their levels through meta-analysis showed limitations in studies (a lack of information on nursery phase duration, detailed information of the presence or absence of additives in diets, complete feed nutritional composition, and, in particular, a lack of Zn-level information). Such limitations may be partly responsible for the high heterogeneity between studies. Future research should explore the modulation of minerals and amino acids in diets, gut health responses, and microbial modulation during nutritional challenges. This would help us to develop a better understanding of the effects of Cu on the performance of nursery piglets and would help guide the rational use of sources and levels of zinc and copper in pig farming.

## 5. Conclusions

Dietary copper supplementation influenced the weight gain and feed conversion rate in weaned piglets, particularly during the first few weeks post-weaning. Copper supplementation levels between 81 and 200 mg Cu/kg improved growth performance, but no further benefits were obtained with higher levels. Such effects were independent of Cu sources, which did not differ between them for any of the studied parameters. High Zn levels in copper diets impair piglet performance.

## Figures and Tables

**Table 1:** Nutritional composition and adjusted means of experimental diets for nursery piglets supplemented with different sources and levels of copper.

Nutrient ^1^	Mean	Minimum	Maximum
DE, kcal/kg	3388.0	3071.6	3843.1
ME, kcal/kg	3240.5	2947.6	3666.3
CP, %	20.0	11.67	25.29
Ca, %	0.85	0.134	1.64
P total, %	0.70	0.25	0.94
Ca:P	2.26	0.40	4.50
Lys, %	1.22	0.79	1.88
Met, %	0.34	0.25	0.70
Met + Cys, %	0.64	0.31	0.97
Thr, %	0.80	0.48	0.97
Trp, %	0.22	0.13	0.30
Copper, mg/kg	120.9	5.00	1500
Zinc, mg/kg	665.4	45.0	3125
Zn:Cu	13.92	0.05	250

^1.^ DE: digestible energy, ME: metabolizable energy, Ca: calcium, CP: crude protein, P total: phosphorus total, Lys: lysine, Met: methionine, Met + Cys: methionine and cystine, Thr: threonine, Trp: tryptophan, Zn: zinc; Cu: copper.

**Table 2:** Effect of copper supplementation on growth performance of nursery pigs.

	N ^1^	ADFI ^2^, kg/d	ADG ^3^, kg/d	F: G ^4^
Copper Sources				
Basal	194	0.561 ± 0.08	0.344 ± 0.05 ^a^	1.65 ± 0.03 ^a^
Inorganic	429	0.596 ± 0.09	0.374 ± 0.06 ^b^	1.61 ± 0.03 ^b^
Organic	323	0.589 ± 0.08	0.372 ± 0.06 ^b^	1.58 ± 0.02 ^b^
Copper levels				
1–15 mg/kg	194	0.597 ± 0.09	0.343 ± 0.06 ^a^	1.70 ± 0.02 ^a^
16–80 mg/kg	130	0.595 ± 0.09	0.363 ± 0.05 ^a^	1.64 ± 0.03 ^a^
81–200 mg/kg	429	0.584 ± 0.08	0.375 ± 0.05 ^b^	1.57 ± 0.03 ^b^
>201 mg/kg	125	0.586 ± 0.12	0.384 ± 0.07 ^b^	1.54 ± 0.04 ^b^
Effects		*p*-value ^5^		
Sources		0.663	0.013	0.020
Level		0.166	0.005	<0.001
Sources * Level		0.478	0.210	0.515
Weaning Age, d		<0.001	<0.001	0.005
Initial body weight, kg		<0.001	<0.001	<0.001

^1^ N = number of observations of treatments. ^2^ ADFI: average daily feed intake; ^3^ ADG: average daily gain; ^4^ F:G: feed to gain ratio. * Least squares means adjusted by covariate to weaning age. ^a,b^ Means in a column followed by no common letter differ significantly (*p* < 0.05). ^5^ *p*-value: significance level of α = 0.05 was considered.

**Table 3:** Multiple regression using mixed models to evaluate the efficiency of supplementation with copper sources and levels on nursery piglet performance.

	ADFI (kg/d)	ADG (kg/d)	F: G
	Model 1 (SE)	Model 2 (SE)	Model 3 (SE)
Intercept	−0.0059 (0.0284)	0.0433 (0.0187) *	1.381 (0.0369) ***
Ingested Cu, mg/d	0.0022 (0.0004) ***	0.0013 (0.003) ***	0.00002 (0.0004) *
Ingested Zn, mg/d	0.0003 (0.00005) ***	0.00001 (0.00011) ***	0.00007 (0.00005)
Zn/Cu ratio	0.00005 (0.00017)	−0.00001(0.00001)	0.00005 (0.0003)
Initial BW, kg	0.02681 (0.0027) ***	0.0128(0.002) ***	0.0154 (0.0032) ***
Initial age	0.0107 (0.0010) ***	0.006 (0.0006) ***	0.00297 (0.0012) *
Inorganic Cu	0.0222 (0.0157)	−0.0024(0.009)	0.07437 (0.0182) ***
Organic Cu	0.0084 (0.0132)	0.00352(0.008)	0.0179 (0.0153)
AIC ^1^	−1600.6	−798.3	−539.1
BIC	−1553.3	−751.1	−491.9
Observations	834	834	834
Variance	0.01	0.02	0.04
Residual variance	0.01	0.02	0.02
Conditional R2	0.737	0.735	0.654
Marginal R2	0.365	0.455	0.080
RMSE	0.081	0.133	0.153
ICC	0.586	0.514	0.623

ADFI: average daily feed intake; ADG: average daily gain; F:G: feed to gain ratio. ^1^ AIC: Akaike information criteria; BIC: Bayesian information criteria; R^2^: determination coefficient; RMSE: root mean square error; ICC: intraclass correlation coefficient. Significance level * (*p* < 0.05); *** (*p* < 0.001).

## Data Availability

The original data presented in the study are included in the article/supplementary material, further inquiries can be directed to the corresponding author/s.

## Supplementary Materials

The following supporting information can be downloaded at: https://www.mdpi.com/article/10.3390/vetsci11020068/s1, Table S1: Database used in meta-analysis approach of copper fonts and levels supplemented in nursery piglet’s diets [10,11,29,30,41,42,43,44,45,46,47,48,49,50,51,52,53,54,55,56,57,58,59,60,61,62,63,64,65,66,67,68,69,70,71,72,73,74,75,76,77,78,79,80,81,82,83,84,85,86,87,88,89,90,91,92,93,94,95,96].

## References

[1] Liao P., Shu X., Tang M., Tan B., Yin Y. (2018). Effect of Dietary Copper Source (Inorganic vs. Chelated) on Immune Response, Mineral Status, and Fecal Mineral Excretion in Nursery Piglets. Food Agric. Immunol..

[2] Dalto D.B., Da Silva C.A. (2021). A Survey of Current Levels of Trace Minerals and Vitamins Used in Commercial Diets by the Brazilian Pork Industry—A Comparative Study. Transl. Anim. Sci..

[3] National Research Council (2012). Nutrient Requirements of Swine.

[4] Espinosa C.D., Fry R.S., Kocher M.E., Stein H.H. (2020). Effects of Copper Hydroxychloride on Growth Performance and Abundance of Genes Involved in Lipid Metabolism of Growing Pigs. J. Anim. Sci..

[5] Hashimoto A., Kambe T. (2015). Mg, Zn and Cu Transport Proteins: A Brief Overview from Physiological and Molecular Perspectives. J. Nutr. Sci. Vitaminol..

[6] Wen Y., Li R., Piao X., Lin G., He P. (2022). Different copper sources and levels affect growth performance, copper content, carcass characteristics, intestinal microorganism and metabolism of finishing pigs. Anim. Nutr..

[7] Villagómez-Estrada S., Pérez J.F., Darwich L., Vidal A., van Kuijk S., Melo-Durán D., Solà-Oriol D. (2020). Effects of copper and zinc sources and inclusion levels of copper on weanling pig performance and intestinal microbiota. J. Anim. Sci..

[8] (2016). Revision of the Currently Authorised Maximum Copper Content in Complete Feed. EFSA J..

[9] Yang P., Wang H., Zhu M., Ma Y. (2019). Effects of Choline Chloride, Copper Sulfate and Zinc Oxide on Long-Term Stabilization of Microencapsulated Vitamins in Premixes for Weanling Piglets. Animals.

[10] Lin G., Guo Y., Liu B., Wang R., Su X., Yu D., He P. (2020). Optimal Dietary Copper Requirements and Relative Bioavailability for Weanling Pigs Fed Either Copper Proteinate or Tribasic Copper Chloride. J. Anim. Sci. Biotechnol..

[11] Yue X., Hu L., Fu X., Lv M., Han X. (2017). Dietary Chitosan-Cu Chelate Affects Growth Performance and Small Intestinal Morphology and Apoptosis in Weaned Piglets. Czech, J. Anim. Sci..

[12] Lovatto P.A., Lehnen C.R., Andretta I., Carvalho A.D., Hauschild L. (2007). Meta analysis in scientific research: A methodological approach. Rev. Bras. Zootec..

[13] St-Pierre N.R. (2007). Meta-Analyses of Experimental Data in the Animal Sciences. R. Bras. Zootec..

[14] Sauvant D., Letourneau-Montminy M.P., Schmidely P., Boval M., Loncke C., Daniel J.B. (2020). Review: Use and Misuse of Meta-Analysis in Animal Science. Animal.

[15] Sales J. (2013). Effects of Pharmacological Concentrations of Dietary Zinc Oxide on Growth of Post-weaning Pigs: A Meta-analysis. Biol. Trace Elem. Res..

[16] Hauschild L., Lovatto P.A., Carvalho A.A., Andretta I., Lehnen C.R. (2008). Relation of plasma zinc and copper with nutritional components and performance of weanling pigs: A meta-analysis. Rev. Bras. Zootec..

[17] van Kuijk S.J.A., Jacobs M., Smits H.M., Han Y. (2019). The Effect of Hydroxychloride Trace Minerals on the Growth Performance And Carcass Quality of Grower/Finisher Pigs: A Meta-Analysis. J. Anim. Sci..

[18] Ketata M.A., Létourneau-Montminy M., Guay F. (2023). Estimation of Digestible Zinc and Copper in Pigs: A Meta-Analysis Approach. KeAi Anim. Nutr..

[19] Feng C., Xie B., Wuren Q., Gao M. (2020). Meta-Analysis of the Correlation Between Dietary Copper Supply and Broiler Performance. PLoS ONE.

[20] Sauvant D., Schmidely P., Daudin J.J., St-Pierre N.R. (2008). Meta-Analyses of Experimental Data in Animal Nutrition. Animal.

[21] Therrell A., Brady E., Bowers K. Association of American Feed Control Officials Committee Reports. Proceedings of the AAFCO Annual Meeting.

[22] Balduzzi S., Rücker G., Schwarzer G. (2019). How to Perform a Meta-Analysis with R: A Practical Tutorial. Evid. Based Ment. Health.

[23] Higgins J.P.T., Thompson S.G., Deeks J.J., Altman D.G. (2002). Measuring Inconsistency in Meta-Analyses Testing for Heterogeneity. BMJ.

[24] Nakagawa S., Johnson P.C.D., Schielzeth H. (2017). The Coefficient of Determination R2 and Intra-Class Correlation Coefficient from Generalized Linear Mixed-Effects Models Revisited and Expanded. J. R. Soc. Interface.

[25] Moher D., Liberati A., Tetzlaff J., Altman D.G., Antes G., Atkins D., Barbour V., Barrowman N., Berlin J.A., Clark J. (2009). Preferred Reporting Items for Systematic Reviews and Meta-Analyses: The PRISMA Statement. PLoS Med..

[26] de Mello G., Antonio Berto D., Lo Tierzo V., Maria Nascimento Augusto R., Maria Ribeiro da Silva A., Alves da Trindade Neto M., Cordeiro Ensá Junqueira Villela C., Vilela Carneiro Girão L. (2012). Sources of Organic Trace Minerals in Diets for Weaned Piglets. Rev. Bras. Zootec..

[27] Chabaev M.G., Nekrasov R.V., Strekozov N.I., Tsis E.Y., Klementyev M.I. (2020). Effects of Different Levels and Forms of Chelated Metal Proteinates on Productive Performance and Metabolic Processes in Fattening Young Pigs. Russ. Agric. Sci..

[28] Dȩbski B. (2016). Supplementation of Pigs Diet with Zinc and Copper as Alternative to Conventional Antimicrobials. Pol. J. Vet. Sci..

[29] Di Giancamillo A., Rossi R., Martino P.A., Aidos L., Maghin F., Domeneghini C., Corino C. (2018). Copper Sulphate Forms in Piglet Diets: Microbiota, Intestinal Morphology and Enteric Nervous System Glial Cells. Anim. Sci. J..

[30] Huang Y.L., Lloyd K.E., Flowers W.L., Spears J.W. (2015). Effect of Dietary Copper Amount and Source on Copper Metabolism and Oxidative Stress of Weanling Pigs in Short-Term Feeding. J. Anim. Sci..

[31] Xing C., Hao C., Liu L., Xu C., Kuang H. (2014). A Highly Sensitive Enzyme-Linked Immunosorbent Assay for Copper(II) Determination in Drinking Water. Food Agric. Immunol..

[32] Mayorga E.J., Kvidera S.K., Horst E.A., Al-Qaisi M., Dickson M.J., Seibert J.T., Lei S., Keating A.F., Ross J.W., Rhoads R.P. (2018). Effects of Zinc Amino Acid Complex on Biomarkers of Gut Integrity and Metabolism during and Following Heat Stress or Feed Restriction in Pigs. J. Anim. Sci..

[33] Chen F., Luo Z., Chen G.H., Shi X., Liu X., Song Y.F., Pan Y.X. (2016). Effects of Waterborne Cu Exposure on Intestinal Copper Transport and Lipid Metabolism of Synechogobius Hasta. Aquat. Toxicol..

[34] Mendonça M.V., Nakasone D.H., Martinez C.H.G., Gemelli J.L., Pereira A.S.C., Pugine S.M.P., De Melo M.P., Andrade A.F.C., Araújo L.F., Augusto K.Z. (2021). Copper and Zinc Hydroxychloride Cosupplementation Improve Growth Performance and Carcass and Reduce Diarrhea Frequency in Grower-Finisher Pigs. Transl. Anim. Sci..

[35] Espinosa C.D., Stein H.H. (2021). Digestibility and Metabolism of Copper in Diets for Pigs and Influence of Dietary Copper on Growth Performance, Intestinal Health, and Overall Immune Status: A Review. J. Anim. Sci. Biotechnol..

[36] Fry R.S., Ashwell M.S., Lloyd K.E., O’Nan A.T., Flowers W.L., Stewart K.R., Spears J.W. (2012). Amount and Source of Dietary Copper Affects Small Intestine Morphology, Duodenal Lipid Peroxidation, Hepatic Oxidative Stress, and MRNA Expression of Hepatic Copper Regulatory Proteins in Weanling Pigs. J. Anim. Sci..

[37] Faccin J.E.G., Tokach M.D., Goodband R.D., Derouchey J.M., Woodworth J.C., Gebhardt J.T. (2023). Industry Survey of Added Vitamins and Trace Minerals in U.S. Swine Diets. Transl. Anim. Sci..

[38] Byrne L., Murphy R.A. (2022). Relative Bioavailability of Trace Minerals in Production Animal Nutrition: A Review. Animals.

[39] Dalto D.B., Audet I., Roy C., Kétilim Novais A., Deschêne K., Goulet K., Matte J., Lapointe J. (2023). Effects of dietary zinc oxide levels on the metabolism of zinc and copper in weaned pigs. J. Anim. Sci..

[40] Dalto D.B., Audet I., Jacques Matte J. (2019). Impact of Dietary Zinc: Copper Ratio on the Postprandial Net Portal Appearance of These Minerals in Pigs. J. Anim. Sci..

[41] Apgar G.A., Kornegay E.T., Lindemann M.D., Notter D.R. (1995). Evaluation of Copper Sulfate and A Copper Lysine Complex as Growth Promoters for Weanling Swine. J. Anim. Sci..

[42] Armstrong T.A., Spears J.W., Van Heugten E., Engle T.E., Wright C.L. (2004). Effect of Dietary Copper Source (Cupric Citrate and Cupric Sulfate) and Concentration on Growth Performance and Fecal Copper Excretion in Weanling Pigs. J Anim Sci..

[43] Bikker P., Jongbloed A.W., Van Baal J. (2016). Dose-Dependent Effects of Copper Supplementation of Nursery Diets on Growth Performance and Fecal Consistency in Weaned Pigs. J. Anim. Sci..

[44] Capps K.M., Amachawadi R.G., Menegat M.B., Woodworth J.C., Perryman K., Tokach M.D., Dritz S.S., Derouchey J.M., Goodband R.D., Bai J. (2020). Impact of Added Copper, Alone or in Combination with Chlortetracycline, on Growth Performance and Antimicrobial Resistance of Fecal Enterococci of Weaned Piglets. J. Anim. Sci..

[45] Coffey R.D., Cromwelf G.L., Monegue H.J. (1994). Efficacy of a Copper-Lysine Complex as a Growth Promotant for Weanling Pigs. J. Anim. Sci..

[46] Cromwell G.L., Lindemann M.D., Monegue H.J., Hall D.D., Orr D.E. (1998). Tribasic Copper Chloride and Copper Sulfate as Copper Sources for Weanling Pigs. J. Anim. Sci..

[47] Davis M.E., Maxwell C.V., Brown D.C., De Rodas B.Z., Johnson Z.B., Kegley E.B., Hellwig D.H., Dvorak R.A. (2002). Effect of Dietary Mannan Oligosaccharides And(or) Pharmacological Additions of Copper Sulfate on Growth Performance and Immunocompetence of Weanling and Growing/Finishing Pigs. J. Anim. Sci..

[48] Ding H., Zhang Q., Xu H., Yu X., Chen L., Wang Z., Feng J. (2021). Selection of Copper and Zinc Dosages in Pig Diets Based on the Mutual Benefit of Animal Growth and Environmental Protection. Ecotoxicol. Environ. Saf..

[49] Dove C.R., Ewan R.C. (1990). Effect of Excess Dietary Copper, Iron or Zinc on the Tocopherol and Selenium Status of Growing Pigs. J. Anim. Sci..

[50] Dove C.R., Ewan R.C. (1991). Effect of Vitamin E and Copper on the Vitamin E Status and Performance of Growing Pigs. J. Anim. Sci..

[51] Dove C.R. (1995). The Effect of Copper Level on Nutrient Utilization of Weanling Pigs. J. Anim. Sci..

[52] Espinosa C.D., Fry R.S., Usry J.L., Stein H.H. (2017). Copper Hydroxychloride Improves Growth Performance and Reduces Diarrhea Frequency of Weanling Pigs Fed a Corn-Soybean Meal Diet but Does Not Change Apparent Total Tract Digestibility of Energy and Acid Hydrolyzed Ether Extract. J. Anim. Sci..

[53] Espinosa C.D., Fry R.S., Kocher M.E., Stein H.H. (2020). Effects of Copper Hydroxychloride and Dietary Fiber on Intestinal Permeability, Growth Performance, and Blood Characteristics of Nursery Pigs. Anim. Feed. Sci. Technol..

[54] Federizzi K.C. (2014). Efeito da Suplementação De Complexo Metal-Aminoácido De Zinco, Manganês E Cobre Sobre O Desempenho Zootécnico E Integridade Do Aparelho Locomotor De Suínos. Palotina.

[55] Gonzales-Eguia A., Fu C.M., Lu F.Y., Lien T.F. (2009). Effects of Nanocopper on Copper Availability and Nutrients Digestibility, Growth Performance and Serum Traits of Piglets. Livest. Sci..

[56] Gonzalez-Esquerra R., Araujo R.B., Haese D., Kill J.L., Cunha A.F., Monzani P.S., Lima C.G. (2019). Effect of Dietary Copper Sources on Performance, Gastric Ghrelin-RNA Expression, and Growth Hormone Concentrations in Serum in Piglets. J. Anim. Sci..

[57] Gurgel M.P.L. (2014). Avaliação De Fontes De Cobre Sobre O Desempenho De Leitões De 24 a 70 Dias De Idade. Master Dissertation/Thesis.

[58] Hauschild L., Lovatto P.A., Lehnen C.R., Andretta I., Garcia G.G., Daniel E. (2012). Piglets Feeding with Diets Containing Milk Fermented and Zinc And Copper Organic. Arch. Zoot..

[59] Hedemann M.S., Jensen B.B., Poulsen H.D. (2006). Influence of Dietary Zinc and Copper on Digestive Enzyme Activity and Intestinal Morphology in Weaned Pigs. J. Anim. Sci..

[60] Hill G.M., Cromwell G.L., Crenshaw T.D., Dove C.R., Ewan R.C., Knabe D.A., Lewis A.J., Libal G.W., Mahan D.C., Shurson G.C. (2000). Growth Promotion Effects and Plasma Changes from Feeding High Dietary Concentrations of Zinc and Copper to Weanling Pigs (Regional Study). J. Anim. Sci..

[61] Jiao L.F., Zhang Q.H., Wu H., Wang C.C., Cao S.T., Feng J., Hu C.H. (2018). Influences of Copper/Zinc-Loaded Montmorillonite on Growth Performance, Mineral Retention, Intestinal Morphology, Mucosa Antioxidant Capacity, and Cytokine Contents in Weaned Piglets. Biol. Trace Elem. Res..

[62] Liao P., Li M., Li Y., Tan X., Zhao F., Shu X., Yin Y. (2017). Effects of Dietary Supplementation with Cupreous N-Carbamylglutamate (NCG) Chelate and Copper Sulfate on Growth Performance, Serum Biochemical Profile and Immune Response, Tissue Mineral Levels and Fecal Excretion of Mineral in Weaning Piglets. Food Agric. Immunol..

[63] Liu H., Tang X.P., Fang R.J., Yi F., Zhang C., Yang R.Q., Sun F., Zhou S.Y. (2020). Effects of Copper Amino Acids Complex on Growth Performance and Serum Cu-Zn Sod Activity in Piglets. Pak. J. Zool..

[64] Lima I.A.V., Miyada V.S. (2003). Organic and Inorganic Copper as Growth Promoters of Weanling Pigs. Rev. Bras. Zoot..

[65] Luo X.G., Dove C.R. (1996). Effect of Dietary Copper and Fat on Nutrient Utilization, Digestive Enzyme Activities, and Tissue Mineral Levels in Weanling Pigs. J. Anim. Sci..

[66] Mei S.F., Yu B., Ju C.F., Zhu D., Chen D.W. (2009). Effect of Different Levels of Copper on Growth Performance and Cecal Ecosystem of Newly Weaned Piglets. Ital. J. Anim. Sci..

[67] Ma Y.L., Lindemann M.D., Webb S.F., Rentfrow G. (2012). Evaluation of Trace Mineral Source and Preharvest Deletion of Trace Minerals from Finishing Diets on Tissue Mineral Status in Pigs. Asian-Australas J. Anim. Sci..

[68] Ma Y.L., Zanton G.I., Zhao J., Wedekind K., Escobar J., Vazquez-Añón M. (2015). Multitrial Analysis of the Effects of Copper Level and Source on Performance in Nursery Pigs. J. Anim. Sci..

[69] Martin R.E., Mahan D.C., Hill G.M., Link J.E., Jolliff J.S. (2011). Effect of Dietary Organic Microminerals on Starter Pig Performance, Tissue Mineral Concentrations, and Liver and Plasma Enzyme Activities. J. Anim. Sci..

[70] Mendonça M.V. (2018). Effects of the Association of Different Copper and Zinc Sources in the Diet of Weaned Piglets. Master Dissertation/Thesis.

[71] Mello G., Berto D.A., Lo Tierzo V., Augusto R.M.N., Da Silva A.M.R., Trindade Neto M.A., Villela C.C.V., Girão L.V.C. (2012). Sources of Organic Trace Minerals in Diets for Weaned Piglets. Rev. Bras. Zoot..

[72] Muniz M.H.B., Berto D.A., Hauptli L., Fracarolli C., Trindade Neto M.A., Tamassia L.F.M., Wechsler F.S. (2010). Organic and Inorganic Source of Zinc and Cooper as Growth Promoters for Weanling Piglets. Rev. Bras. Zoot..

[73] Namkung H., Gong J., Yu H., de Lange C.F.M. (2006). Effect of Pharmacological Intakes of Zinc and Copper on Growth Performance, Circulating Cytokines and Gut Microbiota of Newly Weaned Piglets Challenged with Coliform Lipopolysaccharides. Can. J. Anim. Sci..

[74] Okiyama W.H.E. (2017). Influence of Sources and Levels of Copper on the Performance of Weaned Pigs. Master Dissertation/Thesis.

[75] Pastorelli G., Rossi R., Zanardi E., Ghidini S., Corino C. (2014). Two Different Forms and Levels of Cuso4 in Piglet Feeding: Liver, Plasma and Faeces Copper Status. J. Anim. Feed Sci..

[76] Pérez V.G., Waguespack A.M., Bidner T.D., Southern L.L., Fakler T.M., Ward T.L., Steidinger M., Pettigrew J.E. (2011). Additivity of Effects from Dietary Copper and Zinc on Growth Performance and Fecal Microbiota of Pigs After Weaning. J. Anim. Sci..

[77] Possobon R.M. (1991). O Cobre Como Estimulante Do Crescimento De Suínos Em Recria. Master Dissertation/Thesis.

[78] Ren P., Chen J., Hancock D., Vazquez-Añón M. (2021). Interactive Effects of Copper Sources and a High Level of Phytase in Phosphorus-Deficient Diets on Growth Performance, Nutrient Digestibility, Tissue Mineral Concentrations, and Plasma Parameters in Nursery Pigs. Biol. Trace Elem. Res..

[79] Schaaf S. (2017). Effect of Dietary Source and Concentrations of Copper, Manganese, and Zinc on Growth Performance and Immune Response of Nursery Pigs. Master Dissertation/Thesis.

[80] Shelton N.W., Tokach M.D., Nelssen J.L., Goodband R.D., Dritz S.S., Derouchey J.M., Hill G.M. (2011). Effects of Copper Sulfate, Tri-Basic Copper Chloride, and Zinc Oxide on Weanling Pig Performance. J. Anim. Sci..

[81] Shurson G.C., Ku P.K., Waxler G.L., Yokoyama M.T., Miller E.R. (1990). Physiological Relationships Between Microbiological Status and Dietary Copper Levels in the Pig. J. Anim. Sci..

[82] Smith J.W., Tokach M.D., Goodband R.D., Nelssen J.L., Richert B.T. (1997). Effects of the Interrelationship Between Zinc Oxide and Copper Sulfate on Growth Performance of Early-Weaned Pigs. J. Anim. Sci..

[83] Song J., Li Y.L., Hu C.H. (2013). Effects of Copper-Exchanged Montmorillonite, as Alternative to Antibiotic, on Diarrhea, Intestinal Permeability and Proinflammatory Cytokine of Weanling Pigs. Appl. Clay. Sci..

[84] Stansbury W.F., Tribble L.F., Orr D.E. (1990). Effect of Chelated Copper Sources on Performance of Nursery and Growing Pigs. J. Anim. Sci..

[85] Thomaz M.C., Watanabe P.H., Pascoal L.A.F., Assis M.M., Ruiz U.S., Amorim A.B., Silva S.Z., Almeida V.V., Melo G.M.P., Robles-Huaynate R.A. (2015). Inorganic and Organic Trace Mineral Supplementation in Weanling Pig Diets. An Acad. Bras. Cienc..

[86] Veum T.L., Carlson M.S., Wu C.W., Bollinger D.W., Ellersieck M.R. (2004). Copper Proteinate in Weanling Pig Diets for Enhancing Growth Performance and Reducing Fecal Copper Excretion Compared with Copper Sulfate. J. Anim. Sci..

[87] Windisch W.M., Gotterbarm G.G., Roth F.X. (2001). Effect of Potassium Diformate in Combination With Different Amounts and Sources of Excessive Dietary Copper on Production Performance in Weaning Piglets. Arch. Anim. Nutr..

[88] Xia M.S., Hu C.H., Xu Z.R. (2005). Effects of Copper Bearing Montmorillonite on the Growth Performance, Intestinal Microflora and Morphology of Wean-Ling Pigs. Anim. Feed Sci. Technol..

[89] Yang W., Wang J., Liu L., Zhu X., Wang X., Liu Z., Wang Z., Yang L., Liu G. (2011). Effect of High Dietary Copper on Somatostatin and Growth Hormone-Releasing Hormone Levels in the Hypothalami of Growing Pigs. Biol. Trace Elem. Res..

[90] Zhang Y., Zhou J., Dong Z., Li G., Wang J., Li Y., Wan D., Yang H., Yin Y. (2019). Effect of Dietary Copper on Intestinal Microbiota and Antimicrobial Resistance Profiles of Escherichia coli in Weaned Piglets. Front Microbiol..

[91] Zhang Z.F., Cho J.H., Kim I.H. (2013). Effects of Chelated Copper and Zinc Supplementation on Growth Performance, Nutrient Digestibility, Blood Profiles, and Fecal Noxious Gas Emission in Weanling Pigs. J. Anim. Sci. Technol..

[92] Zhao J., Allee G., Gerlemann G., Ma L., Gracia M.I., Parker D., Vazquez-Anon M., Harrell R.J. (2014). Effects of a Chelated Copper as Growth Promoter on Performance and Carcass Traits in Pigs. Asian-Australas J. Anim. Sci..

[93] Zhao J., Harper A.F., Estienne M.J., Webb K.E., Mcelroy A.P., Denbow D.M. (2007). Growth Performance and Intestinal Morphology Responses in Early Weaned Pigs to Supplementation of Antibiotic-Free Diets with an Organic Copper Complex and Spray-Dried Plasma Protein in Sanitary and Nonsanitary Environments. J. Anim. Sci..

[94] Zhou W., Kornegay E.T., Van Laarp H., Swinkelss J.W.G.M., Wong E.A., Lindemann M.D. (1994). The Role of Feed Consumption and Feed Efficiency in Copper-Stimulated Growth. J. Anim. Sci..

[95] Zhou W., Kornegay E.T., Lindemann M.D., Swinkels J.W., Welten M.K., Wong E.A. (1994). Stimulation of Growth by Intravenous Injection of Copper in Weanling Pigs. J. Anim. Sci..

[96] Zhu D., Yu B., Ju C., Mei S., Chen D. (2011). Effect of High Dietary Copper on the Expression of Hypothalamic Appetite Regulators in Weanling Pigs. J. Anim. Feed Sci..

