# Supplementary material for: A Systematic Review and Meta-Analysis of the Effects of Various Sources and Amounts of Copper on Nursery Piglets
Source: Vet Sci. 2024 Feb 2;11(2):68. doi: 10.3390/vetsci11020068 (PMC10892854; doi:10.3390/vetsci11020068)
Supplement: Supplementary file 1 [file vetsci-11-00068-s001.zip › vetsci-2676634-supplementary.pdf]

Table S1. Database used in meta-analysis approach of copper fonts and levels supplemented in nursery piglet's diets [10, 11, 29, 30, 41-96].

Study	Authors	Year	Country	N animal	Source[1]	AAFCO[2]	Level, ppm	Genetic lines[3]	Age Ini, d	BW Ini, kg	Performance data
41	Apgar et al.	1995	USA	176	INO/ORG	SUL/CA	100-200	Crossb	21	8.23	Yes
42	Armstrong et al.	2000	USA	121	INO/ORG	SUL/QMA	10-225	HxLxDxY	20	6.40	Yes
43	Bikker et al.	2016	Netherlands	80	INO	SUL	80-160	LxD	26	7.90	Yes
44	Capps et al.	2020	-	320	INO	SUL	200	DNA®	21	7.40	Yes
45	Coffey et al.	1994	USA	1300	INO/ORG	SUL/CA	100-200	Crossb	21	7.45	Yes
46	Cromwell	1998	USA	635	INO	SUL/CL	100-200	HxY	29	8.63	Yes
47	Davis et al.	2002	USA	216	INO	SUL	20-175	HxLxDxY	18	6.00	Yes
29	Di Giancamillo et al.	2017	Italy	90	INO	SUL	150	LxLW	26	8.40	**No**
48	Ding et al.	2021	China	400	INO	SUL	80-120	DxLxY	24	9.40	Yes
49	Dove and Ewan	1990	USA	64	INO	SUL	5-250	YxLxD	24	7.19	Yes
50	Dove and Ewan	1991	USA	32	INO	SUL	5-225	YxLxD	-	15.6	Yes
51	Dove	1995	USA	90	INO	SUL	250	YxHxD	26	6.82	Yes
52	Espinosa et al.	2017	USA	80	INO	CL	14-171	PIC®	-	6.80	Yes
53	Espinosa et al.	2020	USA	32	INO	CL	150	PIC®	28	8.33	Yes
54	Federizzi et al.	2014	Brazil	2880	ORG	CA	30	LxLW	23	6.95	Yes
55	Gonzalez-Eguia et al	2009	China	115	INO	SUL	17-50	YxLxD	28	9.63	Yes
56	Gonzalez-Esquerra et al.	2005	Brazil	256	INO/ORG	SUL/QMA/TBBC	160	PIC®	24	6.13	Yes
57	Gurgel et al.	2014	Brazil	96	INO/ORG	SUL/CA	150-580	PIC®	24	3.91	Yes
58	Hauschild	2012	Brazil	64	ORG	CA	200-250	Crossb	21	6.73	Yes
59	Hedemem et al.	2006	Denmark	32	INO	SUL	175	DLxY	28	8.20	Yes
60	Hill et al.	2000	USA	1365	INO	SUL	15-266	-	22	6.55	Yes
30	Huang et al.	2015	China	48	INO	SUL/TBBC	225	-	21	7.03	Yes
61	Jiao et al.	2018	China	108	INO	SUL	75	DLxY	21	6.36	Yes
62	Liao et al.	2017	China	18	INO/ORG	SUL/CA	160	Crossb	28	6.34	Yes
10	Lin et al	2020	China	840	INO/ORG	TBBC/PT	5-160	DxLxY	28	7.38	Yes
63	Liu et al.	2020	China	288	INO/ORG	SUL/CA	100-150	DxLxY	23	8.79	Yes
64	Lima et al.	2003	Brazil	80	INO/ORG	SUL/QMA	50-200	Crossb	21	6.85	Yes
65	Luo and Dove	1996	USA	32	INO/ORG	SUL	15-250	YxHxD	26	6.80	Yes
66	Mei et al.	2009	China	100	INO	CA	10-250	DxLHxY	19	7.50	Yes
67	Ma et al.	2012	USA	-	INO/ORG	SUL/CA	15	YxHxD	21	7.40	Yes

Table S1. Database used in meta-analysis approach of copper fonts and levels supplemented in nursery piglet's diets.

Study	Authors	Year	Country	N animal	Source[1]	AAFCO[2]	Level, ppm	Genetic lines[3]	Age Ini, d	BW Ini, kg	Performance data
68	Ma et al	2015	USA	150	INO/ORG	SUL/CA	125-250	-	21	5.80	Yes
69	Martin et al.	2011	USA	160	INO/ORG	SUL/CA	50-150	YxLxPIC®	35	6.30	Yes
70	Medonça	2018	Brazil	80	INO	SUL	100/3000	-	21	5.68	Yes
71	Mello et al.	2012	Brazil	126	ORG	CA	75-300	-	21	6.10	Yes
72	Muniz et al.	2010	Brazil	-	INO/ORG	SUL/CA	50-240	-	21	5.36	Yes
73	Nankung et al.	2006	Canada	180	INO	SUL	250	Crossb	17	5.90	Yes
74	Okiyama	2017	Brazil	24	INO/ORG	SUL/CL	125-200	-	35	6.10	Yes
75	Pastorelli et al.	2013	Italy	150	INO	SUL	75-150	LxLW	26	8.44	Yes
76-1	Pérez et al.	2011	USA	176	ORG	CA	100	YxLxD	21	5.80	Yes
76-2	Pérez et al.	2011	USA	1008	ORG	CA	100	YxLxD	21	5.20	Yes
76-3	Pérez et al.	2011	USA	120	INO/ORG	SUL/CA	100-250	YxLxD	21	7.00	Yes
76-4	Pérez et al.	2011	USA	-	INO/ORG	SUL/CA	100-315	YxL	21	5,70	Yes
77	Possobon	1991	Brazil	85	INO	SUL	75-300	LHxDxMe	-	9.20	Yes
78	Ren et al.	2021		192		SUL/CA	125	PIC®	-	6.06	Yes
79	Schaaf	2017	USA	280	INO	SUL/CL	50-150	PIC®	20	7.00	Yes
80	Shelton et al.	2011	USA	216	INO	SUL	125-200	-	21	8.79	Yes
81	Shurson et al.	1990	USA	10	INO	SUL	16-283	DxLxY	28	5.50	No
82	Smith et al.	1997	USA	3240	INO	SUL	250	-	17	4.45	Yes
83	Song et al.	2012	China	96	INO	MMT	750-1500	YxLxD	21	5.60	Yes
84	Stansbury et al.	1990	USA	231	INO/ORG	SUL/POL	31,25-250	HxLxDxY	28	6.81	Yes
85	Thomaz et al.	2015	Brazil	70	INO/ORG	SUL/CA	125/250	-	21	6.70	Yes
86	Veum et al.	2004	USA	480	INO/ORG	SUL/PT	25-250	YxLxD	20	6.31	Yes
87	Windish et al.	2001	Germany	96	INO	SUL	25-175	-	28	7.70	Yes
88	Xia et al.	2005	China	128	INO	SUL/MMT	40-150	DxLxY	21	7.50	Yes
11	Yue et al.	2017	China	160	INO/ORG	SUL/POL	100	YxLxD	28	7.74	Yes
89	Yang et al.	2010	China	45	INO/ORG	SUL/CA	125	Crossb	-	7.50	Yes
90	Zhang et al.	2019	Brazil	48	INO	SUL	200	-	21	8.26	Yes
91	Zhang et al.	2013	South Korea	90	ORG	CA/QMA	100	YxL	21	7.84	Yes
92	Zhao et al.	2014	Spain	240	INO/ORG	SUL/POL	6-170	LWXL	26	7.36	Yes
93	Zhao et al.	2007	USA	192	ORG	CA	200-400	DxLxY	18	6.02	Yes

Table S1. Database used in meta-analysis approach of copper fonts and levels supplemented in nursery piglet's diets.

Study	Year	Authors	Country	N animal	Source[1]	AAFCO[2]	Level, ppm	Genetic lines[3]	Age Ini, d	BW Ini, kg	Performance data
94-1	Zhou et al.	1994	USA	42	INO	SUL	15-200	Crossb	21	8.90	Yes
94-2	Zhou et al.	1994	USA	96	INO/ORG	SUL/CA	15-200	Crossb	21	6.81	Yes
95-1	Zhou et al.	1994	USA	144	INO	CL	25-100	YxD	-	6.60	Yes
95-2	Zhou et al.	1994	USA	45	INO	CL	25-	-	-	7.10	Yes
96	Zhu et al.	2011	China	100	INO	SUL	100-250	DxLxY	28	12.29	Yes

[1] INO: Copper Inorganic, ORG: Copper Organic [2] Classified according "The Association of American Feed Control Officials", SUL: Copper Sulfate, CA: Copper complexed with amino acids, POL: Copper complexed with polysaccharides, PT: Copper complexed with proteins, TBBC: Tetrabasic Copper, QMA: Organic metal chelate; [3] S: supranutriotional, R: Requirements. [3] Crossb: crossbreed; D: Duroc; H: Hampshire; L: Landrace; LW: Large White; Me: Meishan; Y: Yorkshire; DNA®: DNA Genetics; PIC®: Pig Improvement Company, Hendersonville, TN.
